# Controversies in intraocular lens implantation in pediatric uveitis

**DOI:** 10.1186/s12348-016-0079-y

**Published:** 2016-03-24

**Authors:** Sumita Phatak, Careen Lowder, Carlos Pavesio

**Affiliations:** Moorfields Eye Hospital NHS Foundation Trust, 162 City Road, London, EC1V 2PD UK; Cleveland Clinic Cole Eye Institute, 9500 Euclid Ave, Cleveland, OH 44106 USA; Inflammation and Immunotherapy Theme, National Institute for Health Research (NIHR) Biomedical Research Centre, Moorfields Eye Hospital NHS Foundation Trust and UCL Institute of Ophthalmology, 162 City Road, London, EC1V 2PD UK

**Keywords:** Pediatric uveitis, Uveitic cataract, Complicated cataract, Intraocular lenses, Chronic uveitis

## Abstract

Cataract is one of the most common and visually debilitating complications of pediatric uveitis. It develops as a consequence of chronic inflammation and steroid use and is seen most often in juvenile idiopathic arthritis (JIA)-associated uveitis. Cataract extraction with intraocular lens (IOL) insertion has been carried out with a measure of success in non-uveitic pediatric eyes, but in cases of uveitis, multiple factors affect the final outcome. Chronic inflammation and its sequelae such as band keratopathy, posterior synechiae, and cyclitic membranes make surgical intervention more challenging and outcome less certain. Postoperative complications like increased inflammation, glaucoma, posterior capsular opacification, retrolental membranes, and hypotony may compromise the visual outcome. Early refractive correction is imperative in pediatric eyes to prevent amblyopia. The use of contact lenses and intraocular lenses in pediatric uveitic eyes were fraught with complications in the past. Surgical interventions such as vitreo-lensectomy followed by contact lens fitting and small incision cataract surgery followed by different types of intraocular lenses have been utilized, and many reports have been published, albeit in small patient groups. This review analyzes and discusses the existing literature on intraocular lens implantation in cases of pediatric uveitic cataract surgery.

## Review

### Introduction

It is particularly important to detect inflammation in the eyes of children because the sequelae not only anatomically change the eye but also, as in the case of pediatric uveitic cataract, lead to sensory amblyopia and squint, reducing the child’s visual potential. Pediatric cataract extraction in non-uveitic eyes has a set of well-established pre-, intra-, and postoperative rules that unfortunately cannot be applied in uveitic cataracts. Pediatric uveitis, especially when due to juvenile idiopathic arthritis (JIA), has a relentless course. The challenges include the control of inflammation so as to reduce anatomical damage, for planning intervention as soon as possible and to reduce postoperative complications. Another very important factor to consider for good visual rehabilitation is best refractive correction. Various techniques like vitreo-lensectomy followed by contact lens fitting, aphakic glasses, and phacoemulsification with intraocular lens insertion have been tried to optimize refractive correction of uveitic eyes with mixed results. To minimize post-surgery inflammatory response, small incision cataract surgery with primary or second-stage intraocular lenses has been studied. Different intraocular lens materials have been tried to test biocompatibility. In this review, we have detailed the published studies on the topic and have attempted to find clarity on the difficult subject of intraocular lens insertion in pediatric uveitic eyes (Table 1).

**Table 1:** Cataract surgery in uveitic eyes and the use of intraocular lenses: comparison of major studies in literature

Sources	No. of patients	Etiology	Age at presentation of Ds in years (median)	Age at Cataract surgery (years)	PCIOL (in the bag) (n =eyes)	Aphakia (n=eyes)	Complications (n=eyes)	Immunosuppression	Follow-up	Comments	Visual outcome pseudophakia (n=eyes)	Visual outcome aphakia (n=eyes)
Probst and Holland [17] (1996)	7 (8 eyes)	JIA		Adults (5) <10 (2)	PMMA		Glaucoma (4), PCO (5)	Steroids	16.6 months		All >20/40	
Lundvall and Zetterstrom [28] (2000)	7 (10 eyes)	JIA		3.5–10 6.5 (mean)	HSM-PMMA 10		Glaucoma (7), PCO (5), 2nd Sx for membranes (8)	Steroids, methotrexate	28 months	Uveitis controlled	20/20–20/50(8) <20/50(2)	
BenEzra and Cohen [15] (2000)	17 (20 eyes)	JIA (8)		4–17	10 PMMA (7), diffractive (3)	7	Glaucoma (4), PS (3), CME (3)	Steroids	60 months	In U/L, CL poorly tolerated, JIA results guarded, uveitis active	JRA 6/9–6/6(1) 6/60(1) 6/240(3)	JRA 6/9–6/6(1) 6/240(3)
Lam et al.[21] (2003)	5 (6 eyes)	JIA (5)	8.5	7–12 8.5 (median)	6 PMMA (4), HSM-PMMA (1), acrylic hydrophobic (1)		PCO (6), glaucoma (2), CME (1)	Methotrexate	43.5 months	Uveitis controlled	ALL >20/40	
Nemet et al. [16] (2007)	18 (19 eyes)	JIA (10), non-JIA (9)	JIA (0.9–14) Non-JIA (4.6–17)	JIA (11.8+/−4.6), non-JIA (17.1+/−4.5)	PMMA (7), acrylic hydrophobic (11), acrylic hydrophilic (1)		PCO (10), glaucoma (4), CME (1), 2nd Sx (11)	Steroids, methotrexate, cyclosporine	45 months	No difference in visual outcome in both groups	>20/40 (13) <20/40 (6)	
Quinones et al. [8] (2009)	34 (48 eyes)	JIA (27), non-JIA (13)	6.7 (4–16)	9.8 (4–17)	13 PMMA	28	Glaucoma (3), PCO (4), RD (3), CME (4), membranes (2)	Steroids, methotrexate	0.3–15.7 years (4.1 years)	Uveitis controlled, postsegment involved (17)	92 % improved >20/40 (8) 20/50–20/70(4) 20/80–20/200(0) <20/400(1)	>20/40 (9) 20/50–20/70(6) 20/80–20/200(5) <20/400(8)
Sijssens [41] (2010)	29 (48 eyes)	JIA	4.4	6.3 aphakia, 7.6 pseudophakia	29 acrylic 24, PMMA 5	19	Ocular HTN, glaucoma, CME, optic disc involvement	Methotrexate	7 years	Uveitis controlled, aphakic surgeries till 2002, complications same in both groups	>20/40 (25) 20/40–20/200(3) <20/200(1)	>20/40 (13) 20/40–20/200(4) <20/200(2)
Terrada et al. [6] (2011)	16 (22 eyes)	JIA (9), non-JIA (7)	5 years	9.5 (median)	22 HSM-PMMA		PCO (2), glaucoma (4), CME (3)	Methotrexate, azathioprine	6.2 years	Uveitis controlled	0.3 or better, log MAR	

*HTN* hypertension, *CME* cystoid macular edema, *RD* retinal detachment, *PS* posterior synechiae, *Sx* surgery

### Inflammation and the pediatric eye

Development of cataract is one of the significant complications of chronic uveitis [1, 2]. Increased susceptibility of a uveitic eye to developing cataract has been attributed in part to a combination of posterior synechiae, systemic corticosteroid therapy, topical corticosteroid therapy exceeding three drops a day, and chronic inflammation [3].

Uveitis presenting in children is significantly different from uveitis in adults in terms of cause as well as presentation. The uveitis of childhood is most commonly seen in patients with JIA who are ANA (antinuclear antibody) positive with oligoarticular arthritis [2, 4, 5]. Other causes of pediatric uveitis are sarcoidosis, pars planitis, and infective etiologies such as toxoplasmosis, toxocariasis, and herpetic infections. A significant proportion is idiopathic [4, 6–8]. Children with JIA–uveitis may remain asymptomatic despite active inflammation, and often, uveitis is detected either during routine screening or because of complications leading to vision loss such as cataract. Different study groups have reported inflammatory sequelae such as band keratopathy, posterior synechiae, cataract, ciliary body inflammation, cyclitic membranes, and reduced aqueous production leading to hypotony in the eyes with chronic inflammation [9, 10].

Over time, protocols have been introduced regarding early screening and aggressive medical treatment of children with uveitis. The American Academy of Pediatrics, British Society for Pediatric and Adolescent Rheumatology, and Royal College of Ophthalmologists have set guidelines for regular screening of JIA patients. It involves high-risk children being screened every 3 months including a detailed ophthalmic evaluation [11–13]. This approach has led to early recognition and better control of inflammation with consequent reduction of inflammatory sequelae.

Reduction of vision due to cataract is of greater relevance in younger children because of the associated risk of amblyopia in this group [9]. Any delay in clearing the visual axis can lead to sensory amblyopia, affecting the eventual visual outcome and quality of life of the child. Unfortunately, results of cataract surgery in children without uveitis cannot be directly applied to uveitic eyes, which have the dual problem of inflammatory sequelae and management of primary cause of inflammation. Tackling both has a profound impact on the timing of surgery as well as eventual visual outcome.

### Factors resulting in poor prognosis and strategies for their prevention

The factors to consider before planning cataract surgery in pediatric uveitic eyes are the following: the etiology of uveitis, patient age, grade of inflammation, preoperative visual acuity, current systemic and local therapy, and risk of amblyopia [8, 14]. Children with JIA–uveitis are likely to have a more complicated post-surgical course compared with uveitis secondary to other causes. This may be because of the younger age at presentation, established inflammatory ocular sequelae, and severe persistent intraocular inflammation [8, 15, 16]. Probst and Holland [17] reported that postoperative complications occur significantly more in children than adults. It is important to know that patients in their study were treated only with steroids and no other additional immunosuppressives.

BenEzra and Cohen [15] studied 10 uveitic postcataract surgery eyes which had undergone intraocular lens (IOL) insertion, in unilateral or asymmetric bilateral cases. The surgery had been performed without waiting for uveitis to completely settle, in order to prevent amblyopia, as the patient age range was 3 to 8 years. No additional systemic steroids were added, and 80 % of patients underwent a second procedure to treat posterior capsular opacification. Terrada et al. [6] recommended intraocular lens insertion as unilateral aphakia led to aniseikonia and amblyopia.

Other than the cataract, factors that can block visual axis or affect preoperative vision are band keratopathy, glaucoma, optic disc involvement, epiretinal membrane, and macular edema [8, 15]. Woreta et al. [9] studied inflammatory sequelae in the eyes with JIA–uveitis and reported band-shaped keratopathy to be the most common (32 %) followed by posterior synechiae (28 %), cataract (22 %), ocular hypertension (15 %), and hypotony (9 %). Epiretinal membrane, optic nerve edema, and macular edema were found in less than 5 % of the eyes. These factors may affect the final visual outcome of these patients. They also observed that 44 % of the eyes with active intraocular inflammation at time of surgery were associated with an almost threefold increase in odds of having postoperative ocular complications.

Cataract surgery causes disruption of blood-aqueous barrier that persists for several weeks in a non-uveitic eye [18]. Eyes with preexisting inflammation have a compromised blood-aqueous barrier, which is responsible for an even greater postoperative disruption of the barrier, leading to fibrin formation and consequent development of inflammatory sequelae. Grajewski et al. [19] reported an improvement of more than two lines in visual acuity after cataract surgery and intraocular lens insertion, in a group of 16 patients. It is significant that surgery had been done only on quiet eyes after good immunosuppression with appropriate topical, systemic steroids, immunosuppressives, and biologicals.

It is important therefore to ensure a two-step process while planning cataract surgery. Step one is to ensure that the inflammation is well controlled before attempting cataract surgery. The mandate is a 3-month inflammation-free period prior to planning cataract surgery [6, 20]. Step two is to continue control in the postoperative period for a good surgical outcome [19, 21]. In recent years, advancement in pharmacotherapy, introduction of biologicals, and intravitreal steroid injections have greatly improved our ability to control inflammation in JIA–uveitis and other forms of pediatric uveitis in a shorter period of time [13, 14, 22]. Hawkins et al. [13] in their review recommended aggressive preoperative and postoperative treatment with topical and systemic steroids, including intravenous methylprednisolone and systemic prednisolone, to achieve bilateral quiet eyes. They recommended the use of immunosuppressive agents such as methotrexate and mycophenolate mofetil in pediatric uveitis for better disease control and as steroid-sparing agents. Cyclosporin was suggested only as an adjunct treatment. Biological agents targeting TNF alpha such as infliximab and adalimumab were recommended, with good results, in uveitis refractory to standard immunosuppressives. Cantarini et al. [22] recommended topical steroids as first-line treatment, adding systemic steroids in cases of failure to achieve control. They warned against long-term systemic steroid use in children and recommend immunosuppressives and biologicals in the same way as Hawkins et al. Grajewski et al. [19] recommended a perioperative intravitreal injection of triamcinolone acetonide in addition to a preoperative well-controlled uveitis for good visual outcomes in JIA–uveitis. Studies conducted on the use of intravitreal steroid implants like Retisert (fluocinolone acetonide) and Ozurdex (dexamethasone) in recalcitrant uveitis in children found that patients uncontrolled on maximum medical therapy benefited by intravitreal introduction of these steroid implants. The eyes needed close monitoring for the development of cataract and glaucoma [23, 24].

### Visual recovery: control of inflammation and refractive correction

Pediatric uveitic eyes needing cataract removal need to overcome multiple triggers of inflammation: the primary cause of uveitis, flare-up from surgical intervention, and the presence of the IOL. Modifications in surgical techniques and testing different IOL materials for minimum immunogenicity have been tried to improved surgical outcome. It is also important to address the challenges faced during biometry for correct IOL power calculation in these eyes.

#### Improvements in surgical techniques

Advances in cataract surgery with smaller incisions and reduced intraoperative manipulation have greatly contributed to reduced inflammation [6, 8, 19, 25]. In the late 1990s, Vasavada et al. advocated performing a primary posterior capsulorhexis with anterior vitrectomy in children with congenital cataract less than 5 years of age [26]. It had been found to reduce the incidence of postoperative retro-IOL membranes and posterior capsular opacification (PCO) and therefore lead to a better visual outcome. Since then, the procedure has been used effectively in surgeries for pediatric uveitic cataract as well [8, 15, 27, 28]. Some studies have reported postsurgical development of PCO and retro-IOL membranes despite this procedure [15, 28], indicating the aggressive nature of inflammation in uveitic eyes. Grajewski et al. [19] suggested performing a pars plana 25-gauge anterior vitrectomy and posterior capsulotomy after phacoemulsification and “in-the-bag” IOL, with reduced incidence of PCO and retrolental membranes.

In situations where the sequelae of inflammation like cystoid macular edema, ciliary membranes, and macular traction do not resolve with medical management, performing a complete vitrectomy with manual removal of the ciliary and intravitreal membranes may assist in relieving associated traction, provided the patient is well supported with good immunosuppression [29]. Palsson et al. [30] reported favorable visual results with combined phacoemulsification, IOL, and vitrectomy in pediatric uveitis but advocated the procedure only for eyes with vitreous pathologies.

Studies in non-uveitic pediatric cataracts reported less postoperative inflammation and complications such as glaucoma when the IOL was placed in the bag [31, 32]. This may be because the lens capsule protects the iris from persistent chafing by IOL haptics. It is seen to benefit uveitic eyes in reducing postoperative inflammation, maybe by decreasing physical contact with uveal structures [8, 16]. Nihalani et al. [31] have used manual separation of the two leaflets of the remaining capsule with a MVR blade for secondary in-the-bag IOL implantation in aphakic non-uveitic pediatric cataract. This may not always be possible in uveitic eyes where postoperative inflammation may lead to fibrosis of the capsule.

Magli et al. [33] suggested that delaying the placement of IOL by about 1 year after cataract extraction significantly reduced secondary glaucoma and retrolental membranes while maintaining similar visual acuity as primary IOL placement in JIA–uveitic cataracts. They suggested a reduced quantum of inflammation as the reason for the better outcome, but, significantly, visual acuities were found to be reduced in sulcus-placed IOLs compared to in-the-bag IOLs. We do not feel that this approach, requiring two interventions and resulting in a sulcus-fixated lens, should be recommended as it may potentially result in UGH syndrome postoperatively.

#### IOL power calculation

Calculating the IOL power is always a challenge in pediatric eyes due to the changing axial lengths and anterior chamber depths of growing eyes, especially in children less than 2 years of age [34]. Children with uveitis, especially JIA–uveitis, usually require cataract surgery around 9.8 years of age (range 4 to 10 years, mean 6 years) [6]. By this age, healthy eyes are sufficiently grown for appropriate IOL measurement. However, in uveitic eyes, there are a number of factors that interfere with surgery planning such as band-shaped keratopathy affecting keratometry readings, posterior synechiae affecting anterior chamber depth, and hypotony and epiretinal membranes affecting axial length measurements. To deal with proper measurement of anterior chamber depth and axial length, immersion A scan biometry seems to be more reliable [35]. Partial coherence interferometry is restricted by the age of the child and density of cataract, especially in uveitic eyes. Anterior segment optical coherence tomography has now become another tool that can help in the accurate determination of anterior chamber depth, but use is limited by a child’s age. All current IOL power calculation formulas have high predictive errors in the shortest eyes. Postoperative refraction target is still controversial, but low degrees of hyperopia do not seem to adversely impact long-term visual acuity in children [34].

#### Refractive correction: IOL or no IOL?

Removing a cataractous lens results in a large refractive error that must be corrected for the best visual outcome. The resulting aniseikonia can result in deep amblyopia, especially in younger children, if not corrected in time. Contact lenses have been used to treat aniseikonia after unilateral cataract surgery. There are many problems that affect the use of contact lenses to correct vision. Eyes with JIA–uveitis are often on long-term topical steroid drops, increasing the chances of developing infective keratitis. Band keratopathy makes contact lens fitting difficult, leading to early intolerance. BenEzra and Cohen [15] found that contact lens was poorly tolerated in their group of patients.

Inserting an intraocular lens (IOL) has been widely accepted for adult uveitic eyes [36] but remains controversial for pediatric uveitic cataracts. In the early 1990s, the use of IOL in pediatric uveitic eyes was associated with significant inflammation, development of intractable glaucoma, cyclitic membrane, hypotony, and phthisis [37, 38]. In these eyes, the degree of fibrosis around the IOL led to cocooning of the IOL in the capsular bag [17, 38]. In 1993, Foster et al.[38] strongly advocated against intraocular lens implantation in children. In retrospect, we can see that these studies did not use systemic immunosuppression to control inflammation.

In the past, surgeons suggested keeping eyes aphakic for better visual outcomes and lensectomies and vitrectomies were procedures of preference in these eyes [38–40], but all of these earlier reports failed to control ocular inflammation and systemic immunosuppressives were not used.

In 1996, Probst and Holland were the first to report on IOL implants in patients with JIA–uveitis. Seven patients (eight eyes) underwent phacoemulsification with intraocular lens insertion, and two were younger than 10 years. A final visual acuity of 20/40 or better was achieved in seven of the eight eyes. Postoperative complications were more common in the two youngest patients, suggesting that intraocular lens implants in younger patients may have more complications [17]. Notably, only corticosteroids were used for suppression of inflammation in their cases.

In 2000, Ben Ezra and Cohen [15] examined the outcomes postcataract surgery with posterior chamber IOL in five eyes of five children (aged 4–8 years) with JIA–uveitis. Three eyes had postoperative visual acuity of 6/240 or less, and complications included posterior synechiae, macular edema, persistent inflammation, and glaucoma. We need to highlight again that the authors had not waited for remission of uveitis before planning cataract surgery, as their focus was the treatment of amblyopia. Peri-operatively, all patients were given retro-orbital methyl prednisolone injections and an intravenous bolus hydrocortisone injection with no additional systemic steroids or immunosuppressives postoperatively. But despite the inflammation, the authors preferred the use of IOLs to contact lens in unilateral aphakia postuveitic cataract extraction [15].

Gradually changing attitudes and better postoperative results were reported in recently published studies and are attributed to improved medical control of inflammation, new surgical techniques, and more biocompatible IOLs [6, 8, 21, 25, 28, 41].

Nemet et al. [16] in a landmark study compared 10 patients with JIA–uveitis with 8 non-JIA–uveitis patients. They observed that eyes with JIA–uveitis had more severe manifestations of uveitis, an earlier presentation of cataract as well as a stormier postsurgical course. They concluded that if inflammation was well controlled, small incision surgery and foldable acrylic hydrophobic IOLs could be well tolerated by these eyes. They also concluded that with effective medical management of inflammation, final visual acuity in patients with JIA–uveitis and of those with other causes of uveitis was comparable. The authors concluded that IOL implantation should no longer be considered an absolute contraindication in pediatric uveitis. We need to note that poor visual outcomes and severe inflammatory sequelae were seen in eyes with poorly controlled inflammation.

Quinones et al. [8] studied the visual outcomes in aphakia and pseudophakia in 34 children (41 eyes) within the age group from 4 to 17 years, with 27 children having JIA-associated uveitis. They reported a 92 % improvement in visual outcome in eyes with polymethyl methacrylate (PMMA) posterior chamber IOLs (PCIOLs) placed in the bag. The authors noted no difference in postoperative inflammation between patients who received an IOL and those who did not. It is important to note that all eyes had been quiet for at least 3 months before surgery, and patients had been given perioperative immunomodulatory therapy. Four patients with JIA received an IOL implant—all four were using methotrexate and received intraocular steroid treatment intraoperatively. Twenty-three percent of children were less than 6 years of age. All of these patients were operated on by C. Stephen Foster who in 1993 recommended against lens implantation in children with JIA.

Similarly, Sijssens et al. [41] compared the outcomes of aphakia versus pseudophakia in a group of 29 children (48 eyes) of JIA-associated uveitis. They noted that though the major risk factors for visual outcome were duration of disease, severity at onset, and age at development of cataract, there was no difference in development of ocular complications in PCIOL and aphakia. They also suggested that with maximum control of inflammation, IOL implantation was associated with better visual outcomes and reduced risk of postoperative CME and glaucoma. They suggested that before surgery, risk of IOL implantation should be evaluated for individual eyes. Shallow anterior chamber, hypotonic eyes, age less than 4 years, and bad outcomes with IOL in fellow eye were considered a contraindication for IOL use.

#### IOL material

As the IOL seems to be the major trigger of the intraocular inflammation in uveitic eyes, various materials have been tried in the search for the least immunogenic one. Studies document the use of different IOL materials like silicone, PMMA, heparin surface-modified PMMA (HSM-PMMA), and hydrophilic and hydrophobic acrylic lenses [6, 28, 40, 42, 43].

In 2004, Ganesh et al. [44] reported a higher incidence of PCO development and inflammatory deposits on PMMA than acrylic lenses. Terrada et al. [6] reported good visual results with HSM-PMMA coated IOLs in children with well-controlled uveitis, within an age group of 4 to 16 years, followed up for a median of 6 years. They went on to suggest that the new generation of foldable acrylic hydrophobic lenses are biocompatible and may reduce inflammatory response because of small incision surgery. Lundvall and Zetterstrom [28] followed up seven children (10 eyes, age range 3.5 to 10 years) postcataract surgery with HSM-PMMA lenses for 5 years. While ensuring uveitis remained under control, they noted that visual acuity improved in nine eyes. They recommended the use of HSM-PMMA lens to correct aphakia, provided the uveitis was well controlled. Similarly, Lam et al. in 2003 studied five patients (six eyes) with well-controlled JIA-associated uveitis who underwent phacoemulsification with IOL (PMMA and acrylic). They advocated the use of IOL in eyes with very good inflammation control, as IOL reduces the risk of amblyopia, while avoiding compliance issues with contact lenses and risk of corneal infections. Though the follow-up period was a median of 43.5 months, the age range was of slightly older children from 7 to 12 years [21].

Alio et al., in their study in adult uveitic cataract, found acrylic IOLs to be associated with least amount of immediate and delayed inflammation, and HSM-PMMA and acrylic IOLs had the least incidence of uveitis relapse. Silicone IOLs had the highest rates of opacification of the posterior capsule [42]. Papaliodis et al., in their study comparing four types of IOL materials, reported acrylic IOLs to be superior to HSM-PMMA, PMMA, and silicone lenses in adult uveitic eyes, when they evaluated inflammation, posterior capsular opacification, visual acuity, and macular oedema [43]. The Perry review concluded that acrylic and HSM lenses performed better in uveitic eyes. They suggested that a lens with a sharp optic edge had better visual outcomes in uveitic eyes because of reduced incidence of PCO [45]. A Cochrane review by Leung et al. on types of intraocular lenses for cataract surgery in uveitis included four studies with a patient profile of adult uveitic eyes [42, 46–49]. They concluded that there was uncertainty as to which type of IOL gave best visual and clinical outcomes in uveitic cataract surgery based on existing studies and advocated a multicenter international study. Abela-Formanek [50], while comparing IOL biocompatibility in 72 uveitic eyes versus 68 control eyes, suggested that though design and biomaterial of the IOL was important, meticulously performed surgery and perioperative management of inflammation could not be overlooked. They compared foldable hydrophilic acrylic, hydrophobic acrylic, and silicone lenses and found hydrophilic acrylic lenses to have good uveal but worse capsular biocompatibility. Hydrophobic acrylic had low uveal but better capsular biocompatibility, and silicone lenses showed more severe anterior capsular contraction. They suggested avoiding round edged hydrophilic acrylic lenses in uveitic eyes because of accelerated rate of PCO formation seen. As all their uveitic patients benefited from the surgery despite the different foldable IOLs, they suggested a longer follow-up to have more conclusive results. Van Gelder et al. [14] in their review for adult uveitis also suggested optimum management of uveitis, including scrupulous attention to preoperative and postoperative inflammation and intraoperative technique for excellent visual outcomes.

## Conclusions

Most of the studies conducted so far on this subject are retrospective, with small groups of patients. The most important prognostic factors for cataract surgery in the eyes of pediatric patients with uveitis have been patient selection and control of intraocular inflammation. All eyes that have done well in published reports have been quiet, well-controlled eyes, with no history of recent flare-ups. Chronic uveitis has been associated with severe complications and poor visual outcomes. Sometimes, in uveitic eyes, especially patients with JIA–uveitis, despite the use of maximum immunosuppressive therapy and biologicals along with local or systemic steroids, the ocular inflammation remains active. This is the group of patients who have poor visual recovery, severe inflammatory sequelae, and sight-threatening complications if an IOL is inserted.

The existing studies advise against using an IOL in patients with active uveitis despite maximum medication, very young children, hypotony, eyes with rubeosis, indeterminate cause of uveitis, and when IOL-related complications have developed in the other eye.

The data advises for minimally invasive small incision cataract surgery with foldable IOL, especially in unilateral cataract, when the uveitis is well controlled and the patient is well controlled systemically,

So far, we do not have definite answers. The existing data are not sufficient for us to derive definitive conclusions or recommendations regarding lens implantation in pediatric uveitis patients. Therefore, multiple factors have to be taken into consideration before deciding for or against IOL insertion in pediatric uveitic eyes. There needs to be a prospective and multicenter study involving ophthalmologists and rheumatologists to have some definite answers on this subject. Till then, patients must be evaluated on a case-by-case basis, using extreme caution, before the final decision is made.

## Abbreviations

ANA: antinuclear antibody
HSM: heparin surface modified
IOL: intraocular lens
PCO: posterior capsular opacification
PMMA: polymethyl methacrylate

## References

[1] Hooper PL, Rao NA, Smith RE (1990). Cataract extraction in uveitis patients. Surv Ophthalmol.

[2] Kesen MR, Setlur V, Goldstein DA (2008). Juvenile idiopathic arthritis-related uveitis. Int Ophthalmol Clin.

[3] Thorne JE, Woreta FA, Dunn JP, Jabs DA (2010). Risk of cataract development among children with juvenile idiopathic arthritis-related uveitis treated with topical corticosteroids. Ophthalmology.

[4] de Boer J, Wulffraat N, Rothova A (2003). Visual loss in uveitis of childhood. Br J Ophthalmol.

[5] Kotaniemi K, Savolainen A, Karma A, Aho K (2003). Recent advances in uveitis of juvenile idiopathic arthritis. Surv Ophthalmol.

[6] Terrada C, Julian K, Cassoux N (2011). Cataract surgery with primary intraocular lens implantation in children with uveitis: long-term outcomes. J Cataract Refract Surg.

[7] Clarke LA, Guex-Crosier Y, Hofer M (2013). Epidemiology of uveitis in children over a 10-year period. Clin Exp Rheumatol.

[8] Quinones K, Cervantes-Castaneda RA, Hynes AY, Daoud YJ, Foster CS (2009). Outcomes of cataract surgery in children with chronic uveitis. J Cataract Refract Surg.

[9] Woreta F, Thorne JE, Jabs DA, Kedhar SR, Dunn JP (2007). Risk factors for ocular complications and poor visual acuity at presentation among patients with uveitis associated with juvenile idiopathic arthritis. Am J Ophthalmol.

[10] Foster CS (2003). Diagnosis and treatment of juvenile idiopathic arthritis-associated uveitis. Curr Opin Ophthalmol.

[11] Cassidy J, Kivlin J, Lindsley C, Nocton J, Section on R, Section on O (2006). Ophthalmologic examinations in children with juvenile rheumatoid arthritis. Pediatrics.

[12] Davies K, Cleary G, Foster H, Hutchinson E, Baildam E, British Society of Paediatric and Adolescent Rheumatology (2010) BSPAR Standards of Care for children and young people with Juvenile Idiopathic Arthritis. Rheumatology(Oxford) 49(7):1406-8 10.1093/rheumatology/kep460 20173199

[13] Hawkins MJ, Dick AD, Lee RJ, et al. (2016) Managing juvenile idiopathic arthritis-associated uveitis. Survey of ophthalmology 61(2):197-201 10.1016/j.survophthal.2015.10.005 26599495

[14] Van Gelder RN, Leveque TK (2009). Cataract surgery in the setting of uveitis. Curr Opin Ophthalmol.

[15] BenEzra D, Cohen E (2000). Cataract surgery in children with chronic uveitis. Ophthalmology.

[16] Nemet AY, Raz J, Sachs D (2007). Primary intraocular lens implantation in pediatric uveitis: a comparison of 2 populations. Arch Ophthalmol.

[17] Probst LE, Holland EJ (1996). Intraocular lens implantation in patients with juvenile rheumatoid arthritis. Am J Ophthalmol.

[18] Sanders DR, Kraff MC, Lieberman HL, Peyman GA, Tarabishy S (1982). Breakdown and reestablishment of blood-aqueous barrier with implant surgery. Arch Ophthalmol.

[19] Grajewski RS, Zurek-Imhoff B, Roesel M, Heinz C, Heiligenhaus A (2012). Favourable outcome after cataract surgery with IOL implantation in uveitis associated with juvenile idiopathic arthritis. Acta Ophthalmol.

[20] Jabs DA, Nussenblatt RB, Rosenbaum JT, Standardization of Uveitis Nomenclature Working G (2005). Standardization of uveitis nomenclature for reporting clinical data. Results of the First International Workshop. Am J Ophthalmol.

[21] Lam LA, Lowder CY, Baerveldt G, Smith SD, Traboulsi EI (2003). Surgical management of cataracts in children with juvenile rheumatoid arthritis-associated uveitis. Am J Ophthalmol.

[22] Cantarini L, Simonini G, Frediani B, Pagnini I, Galeazzi M, Cimaz R (2012). Treatment strategies for childhood noninfectious chronic uveitis: an update. Expert Opin Investig Drugs.

[23] Sella R, Oray M, Friling R, Umar L, Tugal-Tutkun I, Kramer M (2015). Dexamethasone intravitreal implant (Ozurdex(R)) for pediatric uveitis. Graefes Arch Clin Exp Ophthalmol.

[24] Patel CC, Mandava N, Oliver SC, Braverman R, Quiroz-Mercado H, Olson JL (2012). Treatment of intractable posterior uveitis in pediatric patients with the fluocinolone acetonide intravitreal implant (Retisert). Retina.

[25] Hug D (2010). Intraocular lens use in challenging pediatric cases. Curr Opin Ophthalmol.

[26] Vasavada A, Desai J (1997). Primary posterior capsulorhexis with and without anterior vitrectomy in congenital cataracts. J Cataract Refract Surg.

[27] Kotaniemi K, Penttila H (2006). Intraocular lens implantation in patients with juvenile idiopathic arthritis-associated uveitis. Ophthalmic Res.

[28] Lundvall A, Zetterstrom C (2000). Cataract extraction and intraocular lens implantation in children with uveitis. Br J Ophthalmol.

[29] Langner-Wegscheider BJ, de Smet MD (2014). Surgical management of severe complications arising from uveitis in juvenile idiopathic arthritis. Ophthalmologica.

[30] Palsson S, Nystrom A, Sjodell L, et al. (2014) Combined phacoemulsification, primary intraocular lens implantation, and pars plana vitrectomy in children with uveitis. Ocul Immunol Inflamm 23(2):144-51 10.3109/09273948.2014.883546 24564567

[31] Nihalani BR, Vanderveen DK (2011). Secondary intraocular lens implantation after pediatric aphakia. J AAPOS.

[32] Zhu XN, Yu F, Xing XY, Zhao YE, Gong XH, Li J (2013). [Comparison of effects of secondary in-the-bag and sulcus intraocular lens implantation in pediatric aphakia after congenital cataract operation]. Zhonghua Yan Ke Za Zhi.

[33] Magli A, Forte R, Rombetto L, Alessio M. (2014) Cataract management in juvenile idiopathic arthritis: simultaneous versus secondary intraocular lens implantation. Ocul Immunol Inflamm 22(2):133-7 10.3109/09273948.2013.834062 24063263

[34] O’Hara MA (2012). Pediatric intraocular lens power calculations. Curr Opin Ophthalmol.

[35] Wilson ME, Trivedi RH (2012). Axial length measurement techniques in pediatric eyes with cataract. Saudi J Ophthalmol..

[36] Agrawal R, Murthy S, Ganesh SK, Phaik CS, Sangwan V, Biswas J (2012). Cataract surgery in uveitis. Int J Inflamm.

[37] Angeles-Han S, Yeh S (2012). Prevention and management of cataracts in children with juvenile idiopathic arthritis-associated uveitis. Curr Rheumatol Rep.

[38] Foster CS, Barrett F (1993). Cataract development and cataract surgery in patients with juvenile rheumatoid arthritis-associated iridocyclitis. Ophthalmology.

[39] Kanski JJ (1990). Juvenile arthritis and uveitis. Surv Ophthalmol.

[40] Ganesh SK, Sudharshan S (2010). Phacoemulsification with intraocular lens implantation in juvenile idiopathic arthritis. Ophthalmic Surg Lasers Imaging.

[41] Sijssens KM, Los LI, Rothova A (2010). Long-term ocular complications in aphakic versus pseudophakic eyes of children with juvenile idiopathic arthritis-associated uveitis. Br J Ophthalmol.

[42] Alio JL, Chipont E, BenEzra D, Fakhry MA, International Ocular Inflammation Society, SGoUCS (2002). Comparative performance of intraocular lenses in eyes with cataract and uveitis. J Cataract Refract Surg.

[43] Papaliodis GN, Nguyen QD, Samson CM, Foster CS (2002). Intraocular lens tolerance in surgery for cataracta complicata: assessment of four implant materials. Semin Ophthalmol.

[44] Ganesh SK, Babu K, Biswas J (2004). Phacoemulsification with intraocular lens implantation in cases of pars planitis. J Cataract Refract Surg.

[45] Perry LJ, Papaliodis GN (2010). Selection of intraocular lenses in patients with uveitis. Int Ophthalmol Clin.

[46] Mester U, Strauss M, Grewing R (1998). Biocompatibility and blood-aqueous barrier impairment in at-risk eyes with heparin-surface-modified or unmodified lenses. J Cataract Refract Surg.

[47] Roesel M, Heinz C, Heimes B, Koch JM, Heiligenhaus A (2008). Uveal and capsular biocompatibility of two foldable acrylic intraocular lenses in patients with endogenous uveitis—a prospective randomized study. Graefes Arch Clin Exp Ophthalmol.

[48] Tabbara KF, Al-Kaff AS, Al-Rajhi AA (1998). Heparin surface-modified intraocular lenses in patients with inactive uveitis or diabetes. Ophthalmology.

[49] Leung TG, Lindsley K, Kuo IC (2014). Types of intraocular lenses for cataract surgery in eyes with uveitis. Cochrane Database Syst Rev.

[50] Abela-Formanek C, Amon M, Schauersberger J, Kruger A, Nepp J, Schild G (2002). Results of hydrophilic acrylic, hydrophobic acrylic, and silicone intraocular lenses in uveitic eyes with cataract: comparison to a control group. J Cataract Refract Surg.

